# Symmetrically dispersed spectroscopic single-molecule localization microscopy

**DOI:** 10.1038/s41377-020-0333-9

**Published:** 2020-05-25

**Authors:** Ki-Hee Song, Yang Zhang, Benjamin Brenner, Cheng Sun, Hao F. Zhang

**Affiliations:** 1 Department of Biomedical Engineering, Northwestern University, 2145 Sheridan Rd., Evanston, IL 60208 USA; 2 Department of Mechanical Engineering, Northwestern University, 2145 Sheridan Rd., Evanston, IL 60208 USA

**Keywords:** Super-resolution microscopy, Fluorescence spectroscopy

## Abstract

Spectroscopic single-molecule localization microscopy (sSMLM) was used to achieve simultaneous imaging and spectral analysis of single molecules for the first time. Current sSMLM fundamentally suffers from a reduced photon budget because the photons from individual stochastic emissions are divided into spatial and spectral channels. Therefore, both spatial localization and spectral analysis only use a portion of the total photons, leading to reduced precisions in both channels. To improve the spatial and spectral precisions, we present symmetrically dispersed sSMLM, or SDsSMLM, to fully utilize all photons from individual stochastic emissions in both spatial and spectral channels. SDsSMLM achieved 10-nm spatial and 0.8-nm spectral precisions at a total photon budget of 1000. Compared with the existing sSMLM using a 1:3 splitting ratio between spatial and spectral channels, SDsSMLM improved the spatial and spectral precisions by 42% and 10%, respectively, under the same photon budget. We also demonstrated multicolour imaging of fixed cells and three-dimensional single-particle tracking using SDsSMLM. SDsSMLM enables more precise spectroscopic single-molecule analysis in broader cell biology and material science applications.

## Microscopy: Improved localization and analysis of single molecules

Researchers have refined the technique called spectroscopic single-molecule localization microscopy (sSMLM) to simultaneously locate individual molecules and also obtain spectral signatures carrying information about their molecular structures. The method, developed by Hao F. Zhang and colleagues at Northwestern University in Illinois, USA, is the first to use of all of the photons emitted by molecules to provide both spatial and spectral information. Previously the information delivered by photons in separate spatial and spectral channels could not be used in combination. The technique, called Symmetrically dispersed sSMLM, achieves a 42 percent increase in spatial precision and a 10 percent increase in spectral precision. The researchers also demonstrated the technique for multicolour imaging in cells and three-dimensional tracking for monitoring nanoparticles. It should significantly enhance spectroscopic analysis at the single-molecule level in both biology and materials science.

## Introduction

The ability of spectroscopic single-molecule localization microscopy (sSMLM) to capture the spectroscopic signatures of individual molecules along with their spatial distribution allows the observation of subcellular structures and dynamics at the nanoscale. As a result, sSMLM has shown great potential in understanding fundamental biomolecular processes in cell biology and material science^1–7^. It also enables the characterization of nanoparticle properties based on the emission spectrum at the single-particle level^8–10^. Similar to other localization-based super-resolution techniques, such as stochastic optical reconstruction microscopy (STORM) and point accumulation for imaging in nanoscale topography (PAINT), the localization precision of sSMLM is fundamentally limited by the number of collected photons per emitter^11^. However, sSMLM suffers from further photon budget constraints since the collected photons of each molecule need to be divided into two separate channels to simultaneously capture the spatial and spectral information^5–10^. Thus, the spatial localization precision of sSMLM also depends on the splitting ratio between the spatial and spectral channels and is typically limited to 15–30 nm in cell imaging^2,3,5,6^. Although a dual-objective sSMLM design was previously demonstrated with improved spatial localization precision, it imposes a constraint on live-cell imaging and adds complexity to system alignment^1^. The splitting of photons into two channels in sSMLM forces an inherent trade-off between the spatial and spectral localization precisions^5^. Currently, a method to fully utilize the full photon budget to maximize both the spatial and spectral localization precisions in sSMLM is lacking.

To overcome this inherent trade-off, we developed symmetrically dispersed sSMLM, or SDsSMLM, which has two symmetrically dispersed spectral channels instead of one spatial channel and one spectral channel. SDsSMLM fully utilizes all collected photons for both spatial localization and spectral analysis. We showed improvements in the spatial and spectral localization precisions via numerical simulation and validated them by imaging fluorescent nanospheres and quantum dots (QDs). We further demonstrated multicolour imaging of subcellular structures and three-dimensional (3D) single-particle tracking (SPT) capabilities.

## Results

### SDsSMLM

**Fig. 1:**
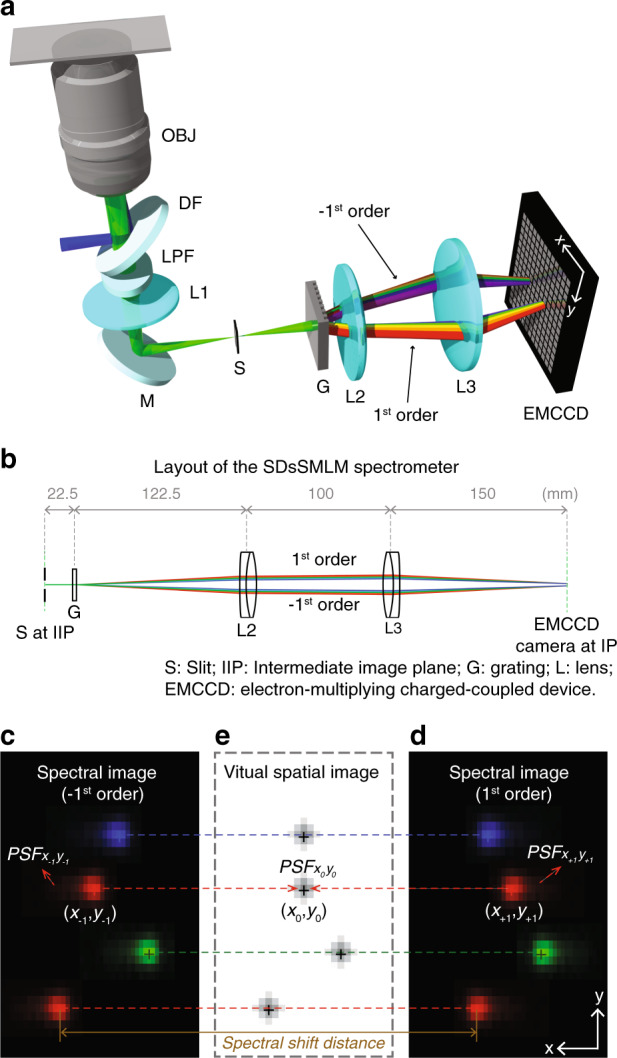
Working principle of SDsSMLM. **a** Schematic of SDsSMLM. **b** Layout of the SDsSMLM spectrometer in Zemax based on the optical components and dimensions used in the experiment. **c** Illustrative image of four molecules from the –1st-order spectral channel. **d** Corresponding image of the same molecules from the 1st-order spectral channel. **e** Virtual spatial image calculated from the two spectral images shown in panels **c** and **d**. OBJ objective lens, DF dichroic filter, LPF longpass filter, L lens, M mirror, S slit, G grating, EMCCD electron-multiplying charge-coupled device.

The concept of SDsSMLM is illustrated in Fig. 1. SDsSMLM is based on a conventional single-molecule localization microscopy (SMLM) system with a grating-based spectrometer (details are described in “Materials and methods” section). In the emission path, the fluorescence light is confined by a slit at the intermediate image plane and symmetrically dispersed into the −1st and 1st orders at an equal splitting ratio by a transmission grating (Fig. 1a). Then, these dispersed fluorescence emissions are captured by an electron-multiplying charge-coupled device (EMCCD) camera to form two symmetrical spectral images after passing through relay optics. In addition, Fig. 1b shows the layout of the SDsSMLM spectrometer in Zemax based on the optical components and dimensions used in our studies.

While existing sSMLM simultaneously captures spatial (0th order) and spectral (1st order) images, SDsSMLM captures only two spectral images (−1st and 1st orders, Fig. 1c, d). The two spectral images of a particular single molecule emission are mirror images of each other with respect to the true location of the molecule. Therefore, we can localize single molecules by identifying the middle points (black plus symbols in Fig. 1e) between the two symmetrically dispersed spectral images. This symmetry–middle point relationship holds true for all molecules regardless of their emission spectra and minute spectral variations even among the same species of molecules. A virtual spatial image can be generated by identifying all the middle points (Fig. 1e). This virtual spatial image utilizes all the detected photons in each EMCCD frame, in contrast to the portion of photons used in existing sSMLM. It should also be noted that the virtual spatial image is not affected by the spectral heterogeneity of individual molecules, which is cancelled out through the symmetry–middle point relationship.

In SMLM, the localization position of individual molecules in the spatial image is estimated with limited certainty^12^. When the localization position is repeatedly estimated, the spatial localization precision (referred to as the spatial precision) is described by the standard deviation of the distribution of the estimated localization positions. Similarly, in SDsSMLM, we estimate the localization positions (*x*_−1_, *y*_−1_) and (*x*_+1_, *y*_+1_) from the −1st order and 1st order spectral images (${\mathrm {PSF}}_{x_{ - 1}y_{ - 1}}$ and ${\mathrm {PSF}}_{x_{ + 1}y_{ + 1}}$ in Fig. 1c, d). Then, we determine the localization position (*x*_0_, *y*_0_) in the virtual spatial image (${\mathrm {PSF}}_{x_0y_0}$ in Fig. 1e) using (*x*_−1_, *y*_−1_) and (*x*_+1_, *y*_+1_), as shown in Fig. 1c–e. Accordingly, the spatial precision in SDsSMLM is described by the standard deviation of the distribution of the estimated (*x*_0_, *y*_0_) in the virtual spatial image (${\mathrm {PSF}}_{x_0y_0}$).

In addition, from the two spectral images (${\mathrm {PSF}}_{x_{ - 1}y_{ - 1}}$ and ${\mathrm {PSF}}_{x_{ + 1}y_{ + 1}}$), we generate new spectral images (${\mathrm {PSF}}_{\lambda y_{ - 1}}$ and ${\mathrm {PSF}}_{\lambda y_{ + 1}}$) based on spectral calibration (details are described in “Materials and methods”section). Then, we integrate them along the *y*-axis and extract spectral centroids (*λ*_SC_) to represent the emission spectra of individual molecules. We calculate *λ*_SC_ as $\lambda _{{\mathrm{SC}}} = \mathop {\sum}\nolimits_\lambda {\lambda I\left( \lambda \right)/\mathop {\sum}\nolimits_\lambda {I\left( \lambda \right)} }$, where *λ* is the emission wavelength and *I*(*λ*) is the spectral intensity at *λ*^13^. Accordingly, the spectral localization precision (referred to as the spectral precision) is described by the standard deviation of the spectral centroid distribution.

Specifically, to generate the virtual image, we first localize the individual molecules in the two spectral images (${\mathrm {PSF}}_{x_{ - 1}y_{ - 1}}$ and ${\mathrm {PSF}}_{x_{ + 1}y_{ + 1}}$) along the *x*-axis using Gaussian fitting based on a maximum-likelihood estimator (MLE)^11,14,15^. Then, we obtain the two localization positions *x*_−1_ and *x*_+1_, which are symmetrically distributed with respect to the true location of the molecule. Therefore, we can determine the spatial location *x*_0_ in the virtual image by calculating the mean of *x*_−1_ and *x*_+1_. In addition, we localize the individual molecules in the two spectral images (${\mathrm {PSF}}_{x_{ - 1}y_{ - 1}}$ and ${\mathrm {PSF}}_{x_{ + 1}y_{ + 1}}$) along the *y*-axis, generating two localization positions *y*_−1_ and *y*_+1_. These localization positions share the same location of the molecule along the *y*-axis. Hence, we can determine the spatial location *y*_0_ in the virtual image by calculating the mean of *y*_−1_ and *y*_+1_.

We can also perform spectral analysis of individual molecules using all the detected photons. We define the distance between *x*_−1_ in Fig. 1c and *x*_+1_ in Fig. 1d as the spectral shift distance (SSD)^10^. Individual molecules with longer emission wavelengths (the red plus symbols in Fig. 1c, d) have larger SSD values than molecules with shorter emission wavelengths (the green and blue plus symbols in Fig. 1c, d). Therefore, we can distinguish individual molecules based on their distinctive SSDs. To obtain the emission spectra of individual molecules, we combine photons from the two spectral images (${\mathrm {PSF}}_{x_{ - 1}y_{ - 1}}$ and ${\mathrm {PSF}}_{x_{ + 1}y_{ + 1}}$) with respect to their spatial locations (*x*_0_, *y*_0_) before spectral fitting, fully utilizing all the collected photons for spectral analysis.

### Single and multicolour SDsSMLM imaging of nanospheres

**Fig. 2:**
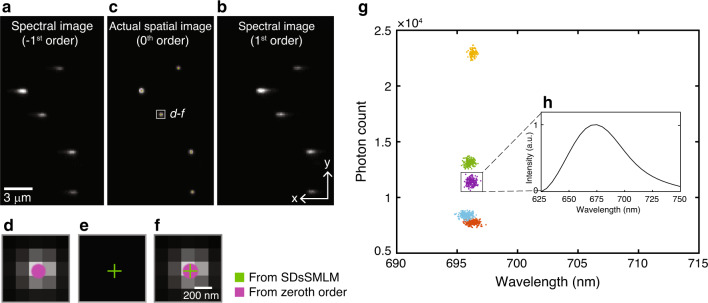
Single-color SDsSMLM imaging of nanospheres. **a**–**c** First frame of the simultaneously captured spectral images and actual spatial image of nanospheres, corresponding to the −1st, 1st, and 0th orders, respectively; **d**, **e** Magnified views to compare the actual and virtual spatial images of the region highlighted by the white box in panel **c**; **f** Corresponding overlaid image; **g** Scatter plot of the photon count versus the spectral centroid; **h** Averaged spectrum of one nanosphere from 200 frames, corresponding to the purple cluster in panel **g**.

To test the feasibility of SDsSMLM, we first imaged fluorescent nanospheres (200-nm diameter, F8807, Invitrogen). As a proof of principle, we used a grating (#46070, Edmund Optics) that split the emitted fluorescence photons into the −1st, 0th, and 1st orders at 22.5%, 28.5%, and 24% transmission efficiencies, respectively (Fig. S1). The −1st order and 1st order images are the symmetrically dispersed spectral images, and the 0th order image is the spatial image. The 0th order image was used for comparison with the virtual spatial image estimated from the −1st and 1st order spectral images. Figure 2a, b show the two symmetrically dispersed spectral images, and Fig. 2c shows the simultaneously captured actual spatial image overlaid with the virtual spatial image. Details of the experiment and the image reconstruction are described in “Materials and methods” section. We observed that the virtual spatial locations (the green plus symbols in Fig. 2c) of nanospheres estimated from the spectral images agree well with the PSFs and further with the directly obtained spatial locations (the magenta circle symbols). The accuracy for the nanosphere in the highlighted region in Fig. 2c is 4.99 nm. (The average accuracy for the five nanospheres is 20.59 nm with a standard deviation of 12.71 nm.) Magnified views of the highlighted region in Fig. 2c are shown in Fig. 2d–f. Note that each localization is rendered using a circle with a 1-pixel diameter for better illustration in Fig. 2d, f. In addition, we numerically corrected the location offset (17.68 ± 23.28 and 32 nm ± 9.99 nm (mean ± standard deviation) along the *x*- and *y*-axes, respectively) between the virtual and actual spatial locations after image reconstruction. In addition, we characterized the accuracy of the SDsSMLM system using a nanohole array. Details of the experiment are described in Supplementary Note 1 and Fig. S2. The accuracy over the entire field of view (FOV) is 14.43 ± 10.25 nm along the *y*-axis and 19.86 ± 12.08 nm along the *x*-axis.

We characterized the spectroscopic signatures of nanospheres using the spectral centroid method^5,13^. Figure 2g shows the scatter plot of the photon count versus spectral centroid for five nanospheres. We observed a narrow spectral centroid distribution of the five nanospheres centred at 696 nm with a spectral precision of 0.35 nm. Figure 2h shows the averaged spectrum of one of the nanospheres from 200 frames (purple cluster in Fig. 2g).

**Fig. 3:**
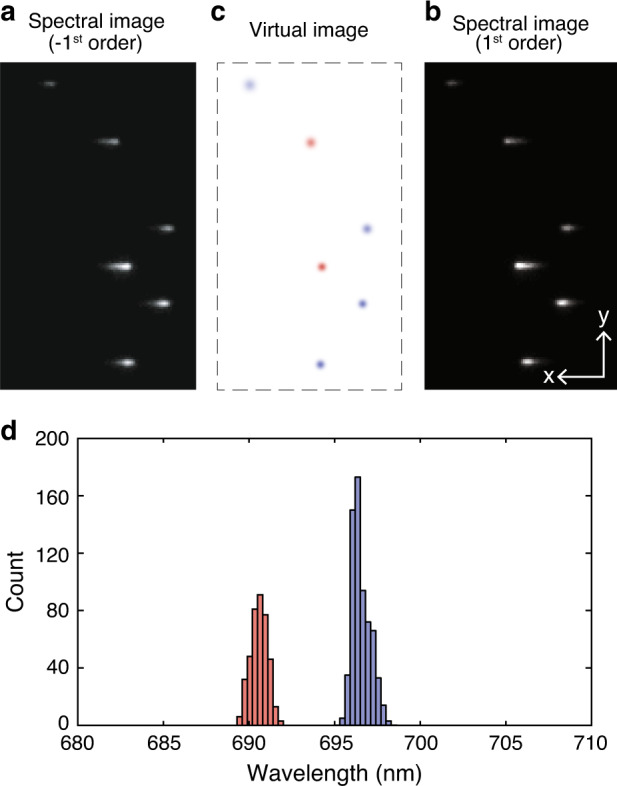
Multi-color SDsSMLM imaging of nanospheres. **a** and **b** First frame of the simultaneously captured spectral images of nanospheres from the −1st and 1st spectral channels, respectively; **c** Calculated virtual spatial image of the nanospheres; **d** Spectral centroid distribution of individual nanospheres. The red and blue colours in panel **c** correspond to the spectral centroids of 690.6 and 696.5nm, respectively, with spectral precisions of 0.48 and 0.53nm.

In addition to functional imaging based on spectral analysis^7^, sSMLM allows multicolour imaging with theoretically unlimited multiplexing capability. The multiplexing capability is predominantly determined by the spectral separation of selected dyes and the spectral precision under given experimental conditions^1,5,6^. We validated this capability of SDsSMLM using two types of nanospheres (200-nm diameter, F8806 and F8807, Invitrogen). Experimental details are described in “Materials and methods” section. Figure 3a, b show the first frame of the simultaneously recorded spectral images. While estimating the spatial locations of individual molecules (Fig. 3c), we successfully classified different types of nanospheres based on their spectral centroid distribution (Fig. 3d). The red and blue colours in Fig. 3c correspond to the spectral centroids of the crimson nanospheres (centred at 690.6 nm with a spectral precision of 0.48 nm) and far-red nanospheres (centred at 696.5 nm with a spectral precision of 0.53 nm), respectively, in Fig. 3d.

### Numerical simulation and experimental validation of the localization precision in SDsSMLM

In SDsSMLM, collected photons are dispersed into more pixels in the spectral image than in the spatial image^5,13^. Thus, the spatial precision of the PSF in spectral images (−1st and 1st orders) is more sensitive to noise contributions than the spatial precision of the PSF in the spatial image (0th order). Such spatial precision is affected not only by the number of collected photons and background but also by experimental parameters in the spectral channel, such as the spectral dispersion (SD)^13^ and full-width at half-maximum (FWHM) of the emission spectrum, which refers to the emission bandwidth of a single molecule. Please see Supplementary Note 2 for details of the SD definition. Through numerical simulation, we investigated the influence of the SD and emission bandwidth of the emission spectrum on the spatial precision, as well as spectral precision under different experimental conditions. We further compared the spatial and spectral precisions in SDsSMLM and sSMLM both numerically and experimentally using QDs. The experimental details are described in “Materials and methods” section.

**Fig. 4:**
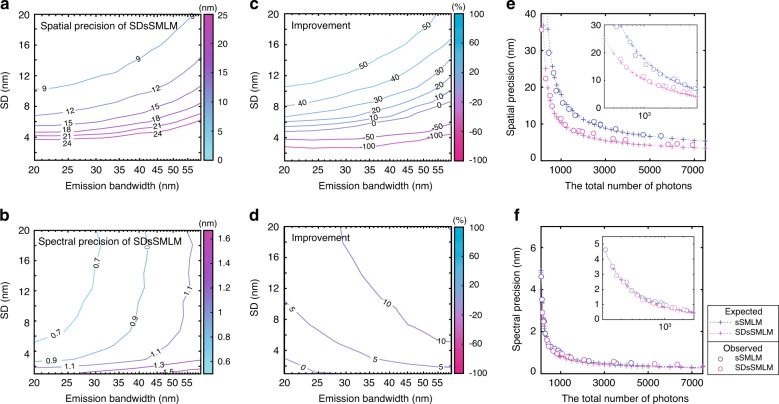
Comparing influences of SD and emission bandwidth on the spatial and spectral precisions between SDsSMLM and sSMLM. **a**, **b** Contour map of the spatial and spectral precisions in SDsSMLM under varying SD and emission bandwidth; **c**, **d** Contour map of improvements in the spatial and spectral precisions in SDsSMLM compared with sSMLM; **e**, **f** Spatial and spectral precisions in SDsSMLM and sSMLM as a function of the number of photons at a 10.5-nm SD and a 35-nm emission bandwidth. The magenta and blue colours represent SDsSMLM and sSMLM, respectively. The plus and circle symbols represent the theoretically and experimentally estimated precisions, respectively.

We compared the achievable spatial and spectral precisions under different SD and emission bandwidth values, where the total photon count was 1000. In sSMLM, we set the splitting ratio between the spatial (0th order) and spectral (1st order) channels to 1:3, following previously reported experimental conditions^2,4–6,8^. We approximated the emission spectrum shape as a Gaussian function. Details of the numerical simulation are described in Supplementary Note 3. Figure 4a shows a 2D contour map of the estimated spatial precision of SDsSMLM. Overall, a larger SD and a narrower emission bandwidth favour higher spatial precision. They also favour higher spectral precision (Figs. 4b and S3b). These trends are fundamentally governed by the contributions of various types of noise, such as the signal shot noise, background shot noise, and readout noise, and they agree well with analytical solutions, especially for the spectral precision^13^. In contrast, sSMLM shows a uniform spatial precision regardless of the SD and emission bandwidth (Fig. S3a). This is because that information in the spectral image is only used for spectral analysis in sSMLM, which is independent from and does not contribute to spatial localization.

Figure 4c, d show the improvements in the spatial and spectral precisions, respectively, in SDsSMLM compared with sSMLM with respect to the SD and emission bandwidth. For example, at a 10.5-nm SD and a 35-nm emission bandwidth, which represent the experimental conditions in imaging QDs, SDsSMLM shows ~42% (from 17.93 to 10.34 nm) and 10% (from 0.90 to 0.81 nm) higher spatial and spectral precisions, respectively, compared with sSMLM. In particular, SDsSMLM offers a relatively uniform improvement, ~10%, in the spectral precision overall. This improvement is proportional to the square root of the ratio of the number of photons allocated to the spectral channel between SDsSMLM and sSMLM (Fig. 4d). We further estimated the achievable spatial and spectral precisions when the number of photons increased. As shown in Fig. 4e, f, the theoretical estimations are in good agreement with the experimental results using QDs. In addition, we investigated the influence of the splitting ratio on the spatial and spectral precisions (Fig. S4).

### Multicolour SDsSMLM imaging of COS7 cells

**Fig. 5:**
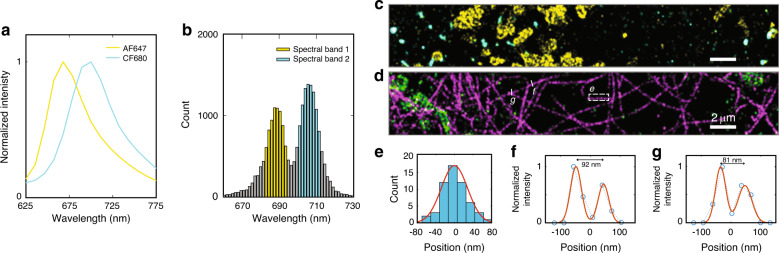
Multi-color SDsSMLM imaging of fixed COS7 cells. **a** Normalized emission spectra of AF647 (yellow) and CF680 (cyan). **b** Spectral centroid distributions of AF647 (yellow, 682–694nm) and CF680 (cyan, 699–711nm). **c** Reconstructed multicolour SDsSMLM image of mitochondria (yellow) and peroxisomes (cyan). **d** Reconstructed multicolour SDsSMLM image of microtubules labelled with AF647 (magenta) and mitochondria labelled with CF680 (green). **e** Histogram of the cross-section highlighted by the white-dashed box in panel **d**. **f**, **g** Intensity profiles of two imaged tubulin filaments highlighted by the white-solid lines in panel **d**.

We demonstrated the multicolour imaging capability of SDsSMLM using fixed COS7 cells. We selected Alexa Fluor 647 (AF647) and CF680, which emit at wavelengths only ~30 nm apart (Fig. 5a), to label mitochondria and peroxisomes, respectively^1,6^. To classify them, we used different spectral bands based on the spectral centroid distribution^5,6^: the first band from 682 to 694 nm for AF647 and the second band from 699 to 711 nm for CF680, as highlighted by the yellow and cyan colours in Fig. 5b, respectively. We visualized the colocalization of mitochondria (yellow) and peroxisomes (cyan) (Fig. 5c). We also imaged microtubules labelled with AF647 (magenta) and mitochondria labelled with CF680 (green) (Fig. 5d). By measuring the FWHM of a segment of an imaged microtubule (dashed square in Fig. 5d), we estimated the spatial resolution of SDsSMLM to be 66 nm, as shown in Fig. 5e. Additionally, we observed that the minimum resolvable distance between two tubulin filaments is within the range of 81–92 nm based on multiple Gaussian fittings of the intensity profiles (Fig. 5f, g). Using the Fourier ring correlation (FRC) method^16^, we also evaluated the resolution of another reconstructed image (Fig. 5c) that visualizes mitochondria and peroxisomes. The FRC curve estimated a resolution of 111 nm (Fig. S5) at a threshold level of 1/7.

In addition, we quantified the utilization ratio, which is defined as the ratio of the number of localizations allocated into each spectral band to the total number of localizations, in the reconstructed image (Fig. 5c). We calculated the utilization ratio in SDsSMLM using both spectral images. We also calculated the utilization ratio by using only one spectral image (1st-order channel), which mimics conventional sSMLM with a 1:1 splitting ratio between the spatial and spectral channels, for comparison. We obtained a 17.4% improvement in the utilization ratio in SDsSMLM, on average for the two spectral channels, compared with that in sSMLM (Fig. S6). This result demonstrates that SDsSMLM benefits from improved spectral precision by fully utilizing all collected photons for spectral analysis, which subsequently leads to improved spectral classification for multicolour imaging.

### 3D single particle tracking

**Fig. 6:**
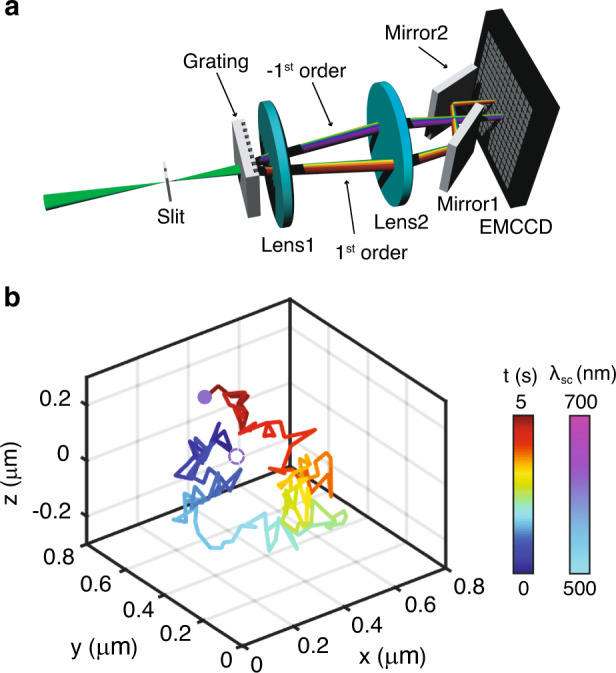
3D biplane SDsSMLM. **a** Schematic of the 3D biplane SDsSMLM system. **b** Imaged 3D trajectory of a single QD colour coded with respect to the acquisition time (solid line). The QDs in the first and last frames are highlighted by circles colour coded with respect to the measured spectral centroids.

We added a 3D imaging capability to SDsSMLM through biplane imaging, similar to what we reported in sSMLM^5^. Since SDsSMLM already has two symmetrically dispersed spectral channels, we can efficiently implement biplane imaging by introducing an extra optical pathlength in one channel. As shown in Fig. 6a, we added a pair of mirrors into the 1st-order spectral channel in front of the EMCCD camera to generate such an optical pathlength difference. This optical pathlength difference introduced a 500-nm axial separation between the imaging planes of the two spectral channels. As a result, individual molecules are imaged with different PSF sizes according to their axial locations. By measuring the ratio between the sizes of the PSFs, we can determine the axial coordinate of each molecule through an axial calibration curve. The full description of biplane SDsSMLM image reconstruction is described in “Materials and methods” section.

We demonstrated 3D biplane SDsSMLM by tracking individual QDs in a suspension. We tracked the movement of QDs for 5 s. We recorded 160 frames with an exposure time of 5 ms at a frame rate of 30 Hz. Figure 6b shows the 3D trajectory of one QD, colour coded with respect to time (represented by the line). The QD locations in the first and last frames are highlighted by the circles colour coded according to the measured spectral centroids. We observed that the spectral centroids remained near 614 nm throughout the tracking period with a spectral precision of 1.5 nm (Fig. S7a). We approximated the diffusion coefficient from the 3D trajectory using *D* = MSD/6*t*, where MSD is the mean squared displacement and *t* is the frame acquisition time^17^. The calculated diffusion coefficient is 0.012 µm^2^/s. These results demonstrate the capability of 3D biplane SDsSMLM to precisely reconstruct the 3D spatial and spectral information of single molecules in SPT.

## Discussion

We demonstrated that SDsSMLM acquires both spatial and spectral information of single molecules from two symmetrically dispersed spectral images without capturing the spatial image. SDsSMLM maintains the highest achievable spectral precision per emitter in given experimental conditions, as it fully uses all collected photons for spectral analysis. In addition, it addresses the inherent trade-off between the spatial and spectral precisions by sharing all collected photons in both spatial and spectral channels. We observed that SDsSMLM achieved 10.34-nm spatial and 0.81-nm spectral precisions with 1000 photons, which correspond to 42%, approximately doubled photon enhancement, and 10% improvements in the spatial and spectral precisions, respectively, compared with sSMLM using a 1:3 ratio between the spatial and spectral channels.

We applied SDsSMLM to multicolour imaging and 3D SPT. It should be noted that these experimental demonstrations were based on a grating that split the beam into the −1st and 1st orders with efficiencies of 22.5% and 24%, respectively. Thus, only approximately half of the photons of the emitted fluorescence were used for image reconstruction in multicolour imaging. Consequently, the current implementation of SDsSMLM has a reduced image resolution. This can be improved by replacing this grating with a new phase grating that can significantly suppress the 0th order and maximize the transmission efficiency only at the −1st and 1st orders, with the relatively high total transmission efficiency expected to be more than 85%^18^. In comparison, the blazed grating reported in our previous sSMLM system^5,6^ has an absolute transmission efficiency of ~18% for the 0th order and an absolute transmission efficiency of ~50% for the 1st order in the far-red channel, corresponding to an overall efficiency of ~68%. Considering the ~85% efficiency of the phase grating, the localization precision will scale favourably due to the increased photon utilization efficiency. In this work, to compare both the spatial and spectral precisions between SDsSMLM and conventional sSMLM, we assumed an identical total number of photons in both systems and 100% absolute transmission efficiency. Specifically, we compared two cases: (1) 25% absolute transmission efficiency for the 0th order and 75% absolute transmission efficiency for the 1st order in the standard sSMLM system and (2) 50% absolute transmission efficiency for both the 1st and −1st orders in SDsSMLM. This reasonably mimics a comparison study using the optimized phase grating and the normal blazed grating. In addition, the resolution can be further improved by using a larger SD and a narrower emission bandwidth, as SDsSMLM favours a large SD and a narrow emission bandwidth for high spatial precision. However, an extremely low SD may compromise one of the benefits of SDsSMLM for functional studies that involve resolving minute spectroscopic features in single-molecule spectroscopy. This suggests that SDsSMLM requires careful dye selection and system optimization to achieve the desired spatial and spectral precisions.

The FOV in SMLM is mainly determined by the objective lens, the field of illumination, and the active area of the camera. For sSMLM equipped with a grating-based spectrometer, the FOV is further restricted by the diffraction angle of the 1st order of the grating, which determines the separation between the spatial and spectral images. In this work, our FOV was restricted to ~30 × 5 µm^2^, as we also captured the 0th order to compare the virtual and actual spatial images. This constraint can be relaxed in the future by using a customized grating that suppresses the 0th order. In this case, the FOV primarily depends on the separation between the −1st and 1st orders, which could increase the FOV by at least two-fold. Additionally, it can be further addressed in 3D biplane SDsSMLM by separately manipulating the two diffraction orders.

In 3D biplane SDsSMLM, the PSFs of the individual molecules in the spectral images are blurred when they are at out of focus planes. This does not allow for a detailed spectral analysis. However, the calculated spectral centroid can still be used to separate two dyes with slightly different fluorescence spectra^5^. In addition, small differences occur in the magnification and the SD between the two spectral images, caused by their different pathlengths. However, the spectral centroid is not significantly affected by these issues and is sufficient for extracting spectroscopic signatures of individual molecules. We numerically corrected them before image reconstruction and spectral analysis. We observe a spectral precision of 1.5 nm throughout the tracking period under the given experimental conditions: the signal level is ~6700 photons, and the background level is ~800 photons in total.

## Materials and methods

### Optical setup and image acquisition for SDsSMLM imaging

We performed all experiments using a home-built SDsSMLM system based on an inverted microscope body (Eclipse Ti-U, Nikon) (Fig. 1a). We used a 640-nm laser to excite nanospheres, AF647, and CF680 and a 532-nm laser to excite QDs. The laser beam was reflected by a dichroic filter (FF538-FDI01/FF649-DI01-25X36, Semrock) and focused onto the back aperture of an oil immersion objective lens (CFI Apochromat ×100, Nikon). We used a high oblique angle to illuminate the samples. The emitted fluorescence light was collected by the objective lens and focused by the tube lens onto the intermediate image plane after passing through a longpass filter (LPF) (BLP01-532R/647R-25, Semrock). We inserted a slit at the intermediate image plane to confine the FOV and subsequently placed a transmission grating (46070, Edmund Optics) to disperse the emitted fluorescence into the −1st, 0th, and 1st orders. Then, the dispersed fluorescence emissions were captured by an EMCCD camera (iXon 897, Andor) with a back-projected pixel size of 160 nm after passing through relay optics (*f* = 150 mm, AC508-150-B-ML, Thorlabs).

For SDsSMLM imaging of nanospheres, we acquired 200 frames at a power density of ~0.02 kW/m^2^ with an exposure time of 20 ms. For the experimental validation of the localization precision using QDs, we acquired 200 frames while varying the signal intensity (photon count) by adjusting the EMCCD exposure time and controlling the illumination power using a neutral density filter (NDC-50C-4M, Thorlabs). For multicolour SDsSMLM imaging of fixed COS7 cells, we acquired 20,000 frames at ~10 kW/cm^2^ with an exposure time of 20 ms. For SPT in 3D, we acquired 160 frames at ~0.02 kW/cm^2^ with an exposure time of 5 ms.

### Image reconstruction for SDsSMLM imaging

For image reconstruction, we first localized the individual molecules in two spectral images (${\mathrm {PSF}}_{x_{ - 1}y_{ - 1}}$ and ${\mathrm {PSF}}_{x_{ + 1}y_{ + 1}}$) with 2D Gaussian fitting using ThunderSTORM^14^. Then, using customized MATLAB codes, we classified them into two groups corresponding to the −1st and 1st orders and estimated the spatial locations of pairs of localizations by calculating their mean values. Next, we formed the virtual image (${\mathrm {PSF}}_{x_0y_0}$) using the estimated spatial locations.

For spectral calibration, we first captured a calibration image using a narrow slit and a calibration lamp. This calibration image included multiple spectral lines of the calibration lamp in the two spectral images. By integrating the two spectral images along the *y*-axis, we obtained emission peaks centred at 487.7, 546.5, and 611.6 nm (Fig. S8a). Then, we obtained a calibration curve by fitting the wavelengths of the emission peaks with their corresponding pixel distances using a linear polynomial function (Fig. S8b). Using the obtained calibration curve, we calibrated the emission spectra of individual molecule pairs. Finally, we obtained the final emission spectra by combining the two symmetrical emission spectra. We used the three emission peaks at 487.7, 546.5, and 611.6 nm of the calibration lamp for the first experimental demonstration using nanospheres and the two emission peaks at 620.23 and 603.24 nm of a neon lamp (6032, Newport) in all other experiments.

To characterize the spectroscopic signatures of individual molecules, we used the spectral centroid^13^. For all the experimental demonstrations, we estimated the spectral centroid in the same manner except for multicolour imaging using nanospheres. Unfortunately, in this experiment, we rarely distinguished two different types of nanospheres based on the spectral centroid values, as the emission of one of the nanospheres (crimson) was partially rejected by the LPF. Thus, we fitted the emission spectrum using a Gaussian function and used the emission peak as an approximation of the spectral centroid.

For imaging nanospheres, QDs, and fixed cells, we used spectral windows of 650–750, 565–665, and 625–775 nm, respectively. In addition, we rejected blinking events below 500 photons during the spectral analysis in multicolour imaging of fixed cells. In addition, we used an SD of 8.8 nm/pixel in nanosphere imaging and an SD of 10.5 nm in all other experiments.

### Image reconstruction for 3D biplane SDsSMLM

We reconstructed the 3D image in a similar manner as previously described for 3D biplane sSMLM^5^ except that we used one symmetrically dispersed spectral image (−1st order), instead of the spatial image (0th order), together with another spectral image (1st order) for biplane imaging. We first captured a 3D calibration image using QDs. This image contained a few samples in both spectral images at different depths. The QDs were scanned from −1.5 to +1.5 µm along the *z*-axis with a step size of 25 nm. Next, we obtained one-dimensional (1D) PSF_y_s by integrating the spectral images along the *x*-axis. Then, we measured the FWHM of the two 1D PSF_y_s and estimated their ratio (Fig. S7b). We used this ratio to calibrate the axial coordinate of each molecule.

### Sample preparation for SDsSMLM imaging

We prepared nanosphere samples for single and multicolour SDsSMLM imaging according to the following steps. Cover glass was rinsed with phosphate buffered saline (PBS), coated with poly-l-lysine (PLL, P8920, Sigma-Aldrich) for 1 h, and washed with PBS three times. Nanospheres (200-nm diameter; F8806 and F8807, Invitrogen) were diluted 10^4^ times with a cross-linking buffer containing EDC (1 mg mL^−1^, 1-ethyl-3-(3-dimethylaminopropyl) carbodiimide hydrochloride) and NHS (1 mg mL^−1^, N-hydroxysuccinimide) in 50 mM MES buffer (2-(N-morpholino) ethanesulfonic acid, pH = ~6, 28390, Thermo Fisher). Two hundred microlitres of the cross-linking buffer with nanospheres was added to the PLL-coated cover glass. The cover glass was rinsed with PBS and dried under filtered air. Then, a drop of antifade mounting medium (P36965, Invitrogen) was added to a cover slip. The cover glass with the samples was mounted on the cover slip by sandwiching the samples between them.

We prepared the QD sample according to the following steps. QDs (777951, Sigma-Aldrich) were diluted 10^4^ times in water. A total of 400 μL of the QD solution with a concentration of 0.5 μg mL^−1^ was deposited onto cover glass using a Laurell WS-650SZ-23NPPB spin-coater at 2000 rpm for 1 min. The cover glass with the sample was mounted on a cover slip by sandwiching the sample between them.

For multicolour SDsSMLM imaging, COS7 cells (ATCC) were maintained in Dulbecco’s modified Eagle medium (DMEM, Gibco/Life Technologies) supplemented with 2 mM l-glutamine (Gibco/Life Technologies), 10% fetal bovine serum (Gibco/Life Technologies), and 1% penicillin and streptomycin (100 U mL^−1^, Gibco/Life Technologies) at 37 °C with 5% CO_2_. Cells were plated on cover glass at ~30% confluency. After 48 h, the cells were rinsed with PBS and then fixed with 3% paraformaldehyde and 0.1% glutaraldehyde in PBS for 10 min at room temperature. After washing with PBS twice, the cells were quenched with 0.1% sodium borohydride in PBS for 7 min and rinsed twice with PBS. The fixed cells were permeabilized with a blocking buffer (3% bovine serum albumin (BSA) and 0.5% Triton X-100 in PBS for 20 min), followed by incubation with the primary antibodies in the blocking buffer for 1 h. For multicolour imaging of mitochondria and peroxisomes, the primary antibodies used in the study were mouse anti-TOM20 directly labelled with AF647 (2.5 μg mL^−1^, sc-17764-AF647, Santa Cruz) and rabbit anti-PMP70 (1:500 dilution, PA1-650, Thermo Fisher). The samples were washed three times with washing buffer (0.2% BSA and 0.1% Triton X-100 in PBS) for 5 min and incubated with secondary antibodies labelled with CF680 (2.5 μg mL^−1^ donkey anti-rabbit IgG-CF680) for 40 min. For multicolour imaging of microtubules and mitochondria, the primary antibodies used in the study were sheep anti-tubulin (2.5 μg mL^−1^, ATN02, Cytoskeleton) and mouse anti-TOM20 (2.5 μg mL^−1^, sc-17764, Santa Cruz). After washing with washing buffer three times for 5 min, the samples were incubated with secondary antibodies labelled with AF647 and CF680 (2.5 μg mL^−1^ donkey anti-sheep IgG-AF647 and anti-mouse IgG-CF680) for 40 min. The dyes were conjugated to the IgG following a literature protocol (degree of label = ~1)^19^. The cells were then washed with PBS three times for 5 min and stored at 4 °C. An imaging buffer (pH = ∼8.0, 50 mM Tris, 10 mM NaCl, 0.5 mg mL^−1^ glucose oxidase (G2133, Sigma-Aldrich), 2000 U/mL catalase (C30, Sigma-Aldrich), 10% (w/v) d-glucose, and 100 mM cysteamine) was used to replace PBS before image acquisition.

For 3D SPT, a QD solution of 0.5 μg mL^−1^ in water was mixed with glycerol (v/v = 1:9) and vortexed for 10 s. Then, 50 μL of the final solution was immediately added onto cover glass. The free-diffusing single QDs were then observed and tracked.

## Supplementary information

Supplementary Information

## Data Availability

The datasets generated and analysed in the article are available from the corresponding author upon reasonable request.
