# Supplementary material for: Symmetrically dispersed spectroscopic single-molecule localization microscopy
Source: Light Sci Appl. 2020 May 25;9:92. doi: 10.1038/s41377-020-0333-9 (PMC7248114; doi:10.1038/s41377-020-0333-9)
Supplement: Supplementary file 1 — Supplementary Information [file 41377_2020_333_MOESM1_ESM.docx]

This document provides supplementary information for

Symmetrically dispersed spectroscopic single-molecule localization microscopy

Ki-Hee Song,^1†^ Yang Zhang,^1†^ Benjamin Brenner,^1^ Cheng Sun,^2^ Hao F. Zhang^1^*

^†^These authors contributed equally to this work

*Corresponding author: hfzhang@northwestern.edu

This file includes:

**Fig. S1**. Schematic of the grating used for the experimental demonstrations.

**Fig. S2**. Estimation of accuracy in SDsSMLM.

**Fig. S3**. Influences of the SD and emission bandwidth of the emission spectrum on the spatial and spectral precisions in sSMLM.

**Fig. S4**. Additional comparisons of the spatial and spectral precisions between SDsSMLM and sSMLM.

**Fig. S5**. FRC curve of the reconstructed multicolour image.

**Fig. S6**. Analysis of the utilization ratio of the reconstructed multicolour image.

**Fig. S7**. 3D SPT.

**Fig. S8**. Spectral calibration information.

**Fig. S9**. Achievable SD in SDsSMLM.

**Fig. S10.** Influence of splitting photons into multiple channels on SNR.

**Supplementary Note 1.** Estimation of accuracy in SDsSMLM.

**Supplementary Note 2.** Achievable SD in SDsSMLM.

**Supplementary Note 3.** Numerical simulation.

**Supplementary Note 4.** Influence of splitting photons into multiple channels on SNR.

**
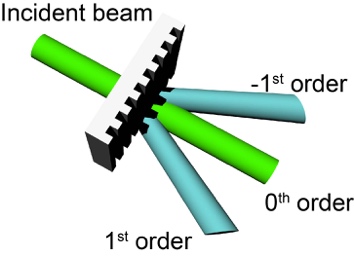
**

**Fig. S1**. Schematic of the grating used for the experimental demonstrations.

**
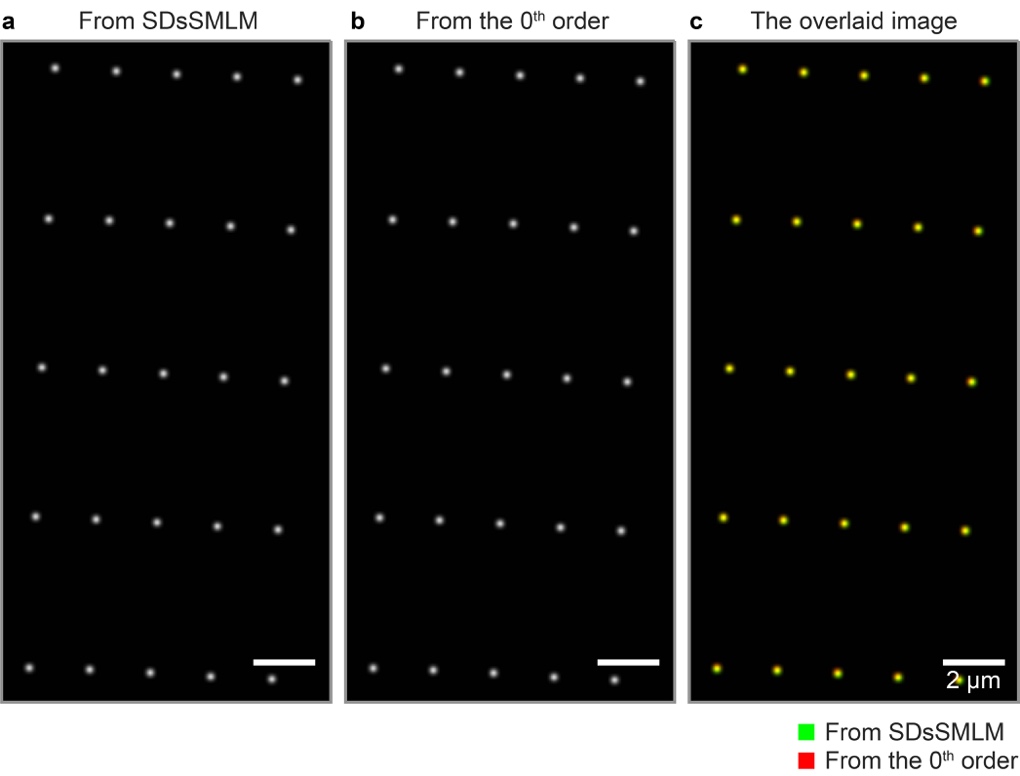
**

**Fig. S2**. Comparison between **a** Reconstructed virtual spatial image and **b** actual 0^th^ order spatial image of the nanohole array. **c** overlaid image of panels a and b.

**
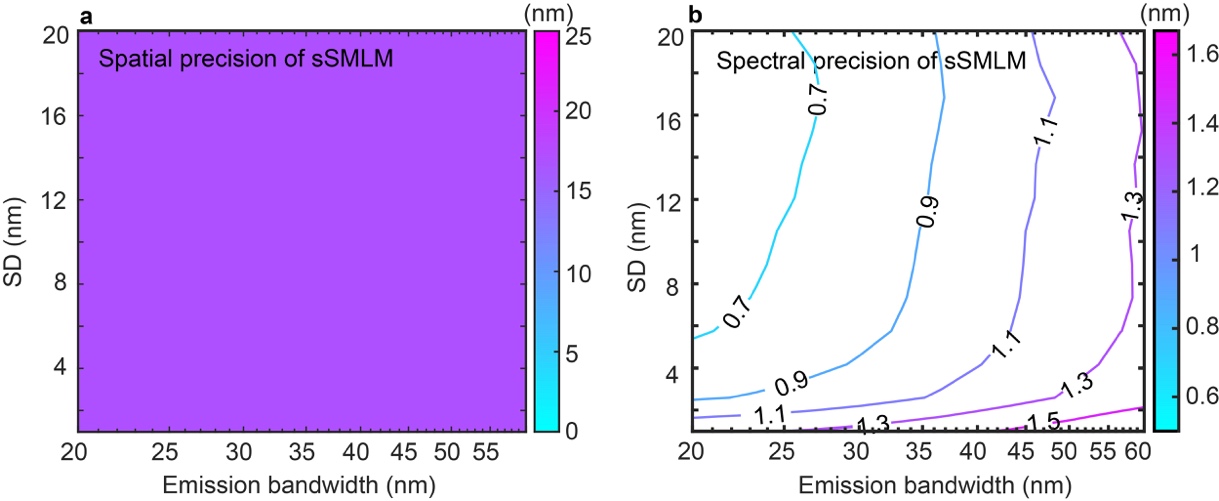
**

**Fig. S3**. Influences of the SD and emission bandwidth of the emission spectrum on the spatial and spectral precisions in sSMLM. **a, b** Contour map of spatial and spectral precisions under varying SD and emission bandwidth.

**
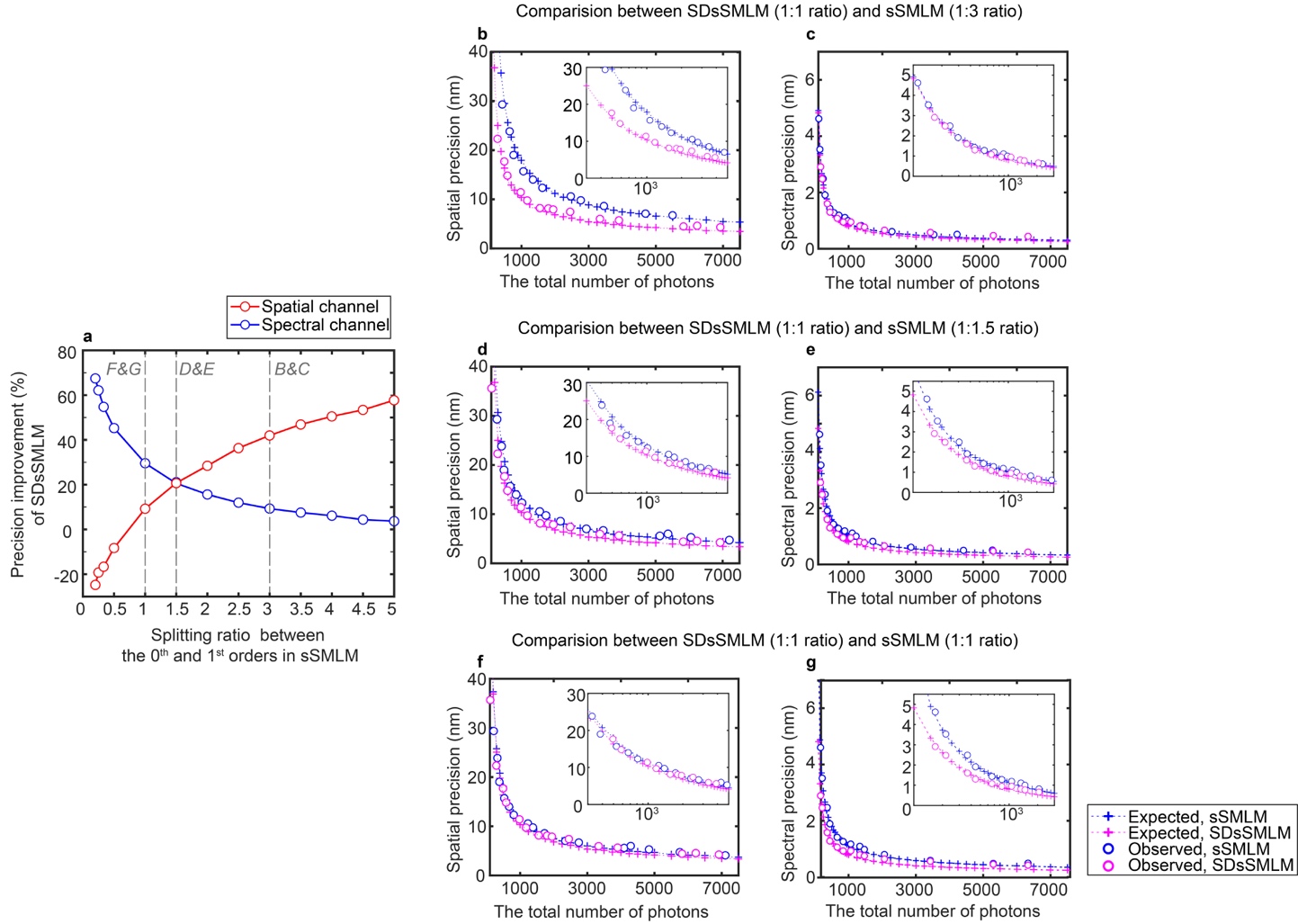
**

**Fig. S4**. Additional comparisons of spatial and spectral precisions between SDsSMLM and sSMLM. **a** Expected precision improvement of SDsSMLM when splitting ratio between the 0^th^ and 1^st^ orders in sSMLM varies. The signal level is 1000 photons. At the splitting ratio of **b**, **c** 1:3, **d**, **e** 1:1.5, and **f, g** 1:1 for sSMLM, the achievable spatial and spectral precisions as a function of the number of photons. SDsSMLM achieves (1) 42 % (from 17.93 nm to 10.34 nm) and 10 % (from 0.90 nm to 0.81 nm) improvements in spatial and spectral precisions, respectively, compared with sSMLM featuring a 1:3 ratio; (2) 19 % spatial (from 12.73 nm to 10.34 nm) and 21 % spectral (from 1.03 nm to 0.81 nm) precision improvements compared to sSMLM with a 1:1.5 ratio; (3) 10 % spatial (from 11.42 nm to 10.34 nm) and 30 % spectral (from 1.15 nm to 0.81 nm) precision improvements compared to sSMLM with a 1:1 ratio.

**
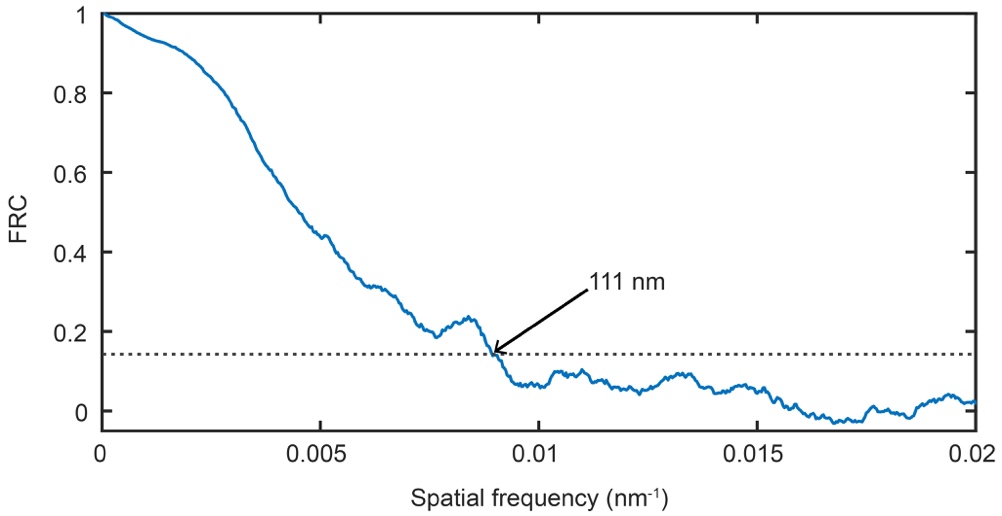
**

**Fig. S5**. FRC curve of the reconstructed multicolour image shown in Fig. 5c.

**
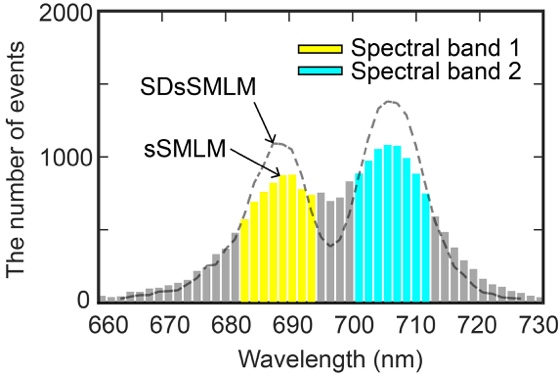
**

**Fig. S6**. Analysis of utilization ratio for the reconstructed multicolour image shown in Fig. 5c. The histogram represents the spectral centroid distribution estimated from only one spectral image corresponding to the 1^st^ order. This case reasonably mimics conventional sSMLM with a 1:1 splitting ratio between the spatial and spectral channels. The dashed line shows the profile of the spectral centroid distribution estimated from two spectral images in the SDsSMLM case. The number of localizations allocated to each spectral band was increased from 6074 to 7119 for the first spectral channel and from 7759 to 9124 for the second spectral channel, which correspond to 17.2 % and 17.6 % improvements in the utilization ratio, respectively.

**
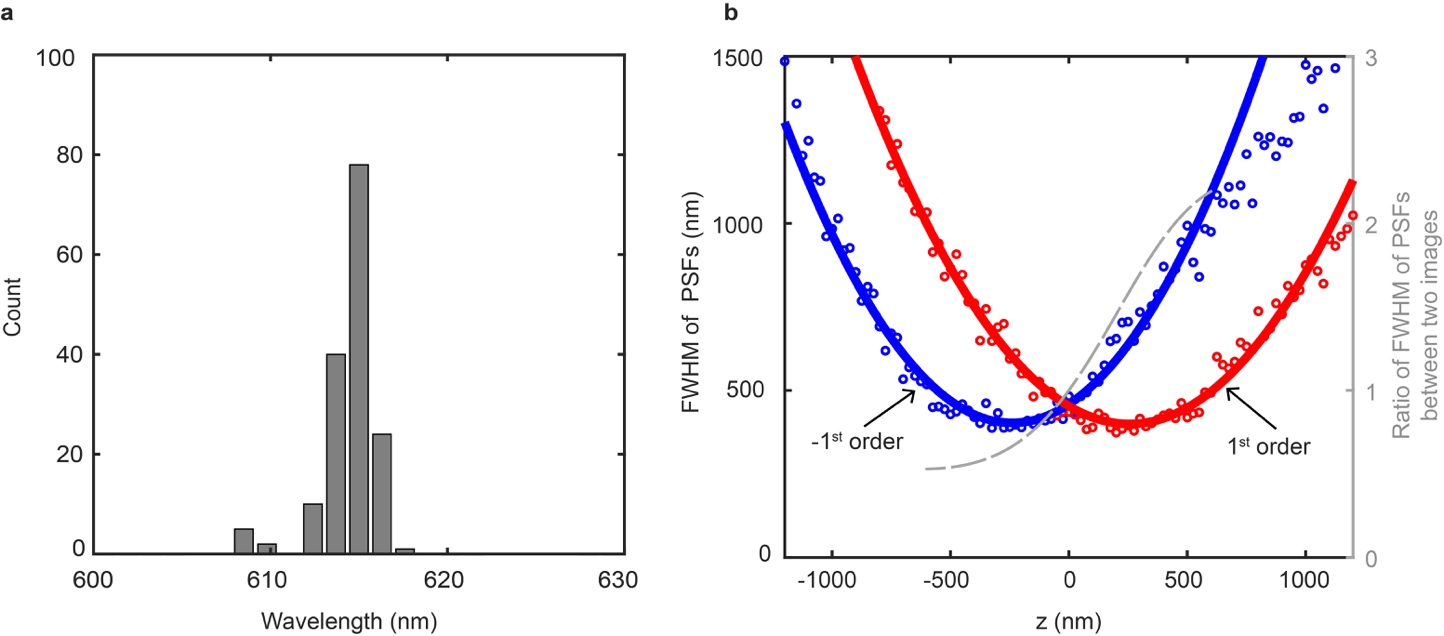
**

**Fig. S7**. **a** Histogram of spectral centroids of the QD during tracking; **b** 3D calibration curve. The blue and red solid lines indicate the FWHM of PSFs in the -1^st^ and 1^st^ orders respectively. The gray dashed line represents the corresponding ratio.

**
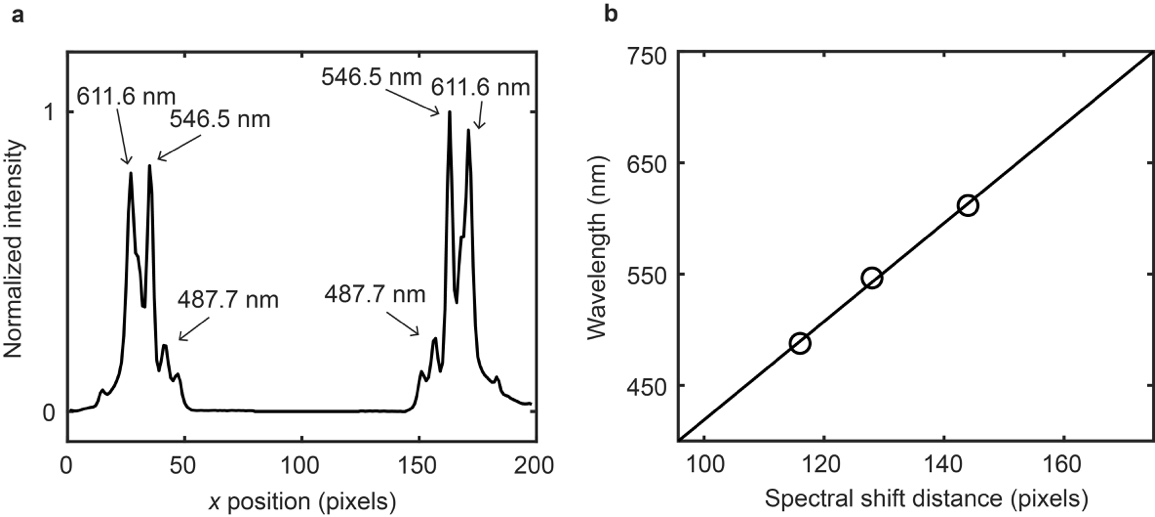
**

**Fig. S8**. Spectral calibration information. **a** Emission peaks of the calibration source centred at 487.7 nm, 546.5 nm, and 611.6 nm; **b** Calibration curve obtained by fitting the wavelengths with their corresponding pixel distances using a linear polynomial function.

**
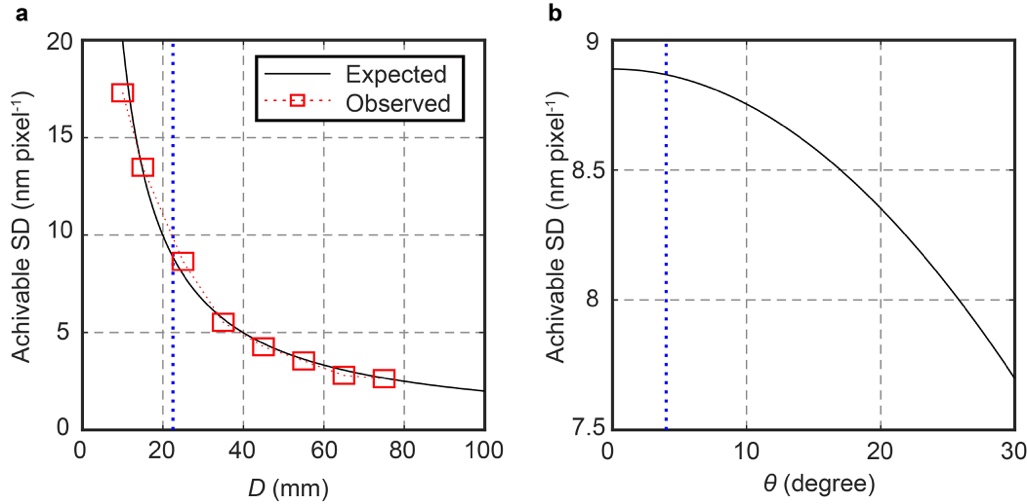
**

**Fig. S9**. Achievable SD values as a function of **a** distance from the intermediate image plane to the grating and **b** diffraction angle of -1^st^ or 1^st^ orders. For estimation, we used experimental conditions: a focal length of 150 mm, a groove density of 80 grooves mm^-1^, a camera pixel size of 16 µm.


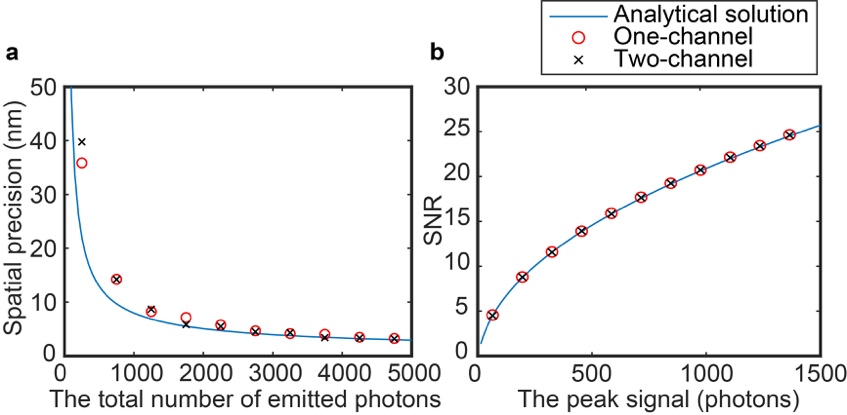


**Fig. S10.** Achievable **a** spatial precision as a function of the total number of emitted photons and **b** SNR as a function of the peak signal in photons for one- and two- channel cases. Key parameters used in the simulation are listed in Table S1.

**Supplementary Note 1. Estimation of accuracy in SDsSMLM**

In SDsSMLM, misalignment and imperfection of the optical system can cause an uncertainty issue in a reconstructed image. This is a well-known issue caused by spherical aberration, field distortion, or asymmetricity of a lens pair with respect to the optical axis. Such imperfections especially influence localization accuracy, which describes the deviation of the mean of the obtained position coordinates from the true position of single emitters, rather than localization precision^1^. Accordingly, we quantified the accuracy of the SDsSMLM system using a custom-made nanohole array with known spacing. We imaged the nanohole array using a white-light lamp and a 532 nm laser line filter with an exposure time of 50 ms. Figs. S2a and S2b show the reconstructed virtual spatial image and the actual 0^th^ order spatial image of the nanohole array, respectively. The image contains 5 holes with spacings of 2 µm along the x-axis and 5 µm along the y-axis, respectively; and the hole size is 200 nm. From the overlaid image (Fig. S2c), we observed that their spatial locations agree with each other reasonably well over the entire field-of-view (FOV). We treat the 0^th^ order image as the ground truth and estimated the difference between Figs. S2a and S2b. The accuracy over the entire FOV were 14.43±10.25 nm along the y-axis and 19.86±12.08 nm along the x-axis. Overall, the lens imperfection led to a localization error of < 20 nm along both axes, which was smaller than the imaging resolution in sSMLM cell imaging using a grating-based spectrometer, typically 40-80 nm^2,3^. In addition, we expect that aberration-induced accuracy issues, in sSMLM, from samples with varying refractive index mismatch can be canceled out and minimized by the symmetricity in SDsSMLM if the accuracy issue caused by the asymmetry of the lenses is negligible.

**Supplementary Note 2. Achievable SD in sSMLM**

Spectral dispersion (SD) is defined as the wavelength range per individual pixel (nm pixel^-1^)^4^. It is mainly determined by the camera pixel size, the diffraction angle at the -1^st^ or 1^st^ orders, and the effective focal length of relay optics. This can be approximated by^5^,

|  | $\Delta\lambda=W_{p}\times\frac{dcos\theta}{f\times m}$, | (S1) |
| --- | --- | --- |

where $W_{p}$ [µm] is the camera pixel size; *d* [µm] is the groove spacing; $\theta$ [degree] is the diffraction angle; *m* [dimensionless] is the order of the maxima of the blazed grating (typically 1); and *f* [mm] is the effective focal length of the relay optics defined by ${{Df}_{2}}/{f_{1}}$. *D* [mm] is the distance from the grating position to the intermediate image plane; $f_{1}$ [mm] is the focal length of the collimating lens (L2 in Fig. 1a); $f_{2}$ [mm] is the focal length of the focusing lens (L3 in Fig. 1a).

As shown in Eq. S1, increasing the diffraction angle ($\theta$) will reduce the achievable spectral dispersion $\Delta\lambda$ (meaning a better resolving ability). Our desired SD in sSMLM, however, is relatively low (typically 3-9 nm pixel^-1^)^3,5,6^, which corresponds to a relatively low diffraction angle (typically < 10 degrees). This can be sufficiently covered by the 2-inch imaging lens used in our experiment.

We quantified the achievable SD as a function of *D* and $\theta$ at given experimental conditions both theoretically and experimentally. As shown in Fig. S9a, we can tune the SD by changing *D*^3,5^. The blue dotted line indicates the approximate distance (22.5 mm) used in our experiment to achieve a SD of 9 nm pixel^-1^. In addition, at *D* = 22.5 mm, we can tune the SD from 7.5 nm pixel^-1^ to 9 nm pixel^-1^ by changing the diffraction angle (Fig. S9b), which is primarily determined by the groove density of a grating and the emission wavelength range.

While the SD can be determined by the grating position according to the Eq. S1, spatial information at the intermediate image plane is preserved and formed at the EMCCD chip, which is a conjugate image plane of the intermediate image plane, regardless of the grating. In this work, the grating position was set to be 22.5 mm from the intermediate image plane, which corresponds to an SD of 9 nm pixel^-1^.

**Supplementary Note 3.** Numerical simulation

To simulate two symmetrical spectral images in symmetrically dispersed spectroscopic single-molecule localization microscopy (SDsSMLM), we first generated a spatial image. The spatial image was modeled as a 2D Gaussian function with a sigma value of 0.94 pixel, which represents the experimental conditions: back-projected pixel size of 160 nm and point spread function (PSF) full-width at half-maximum (FWHM) of 350 nm. Then, we convolved the generated spatial image with the emission spectrum of the dye molecule being simulated to generate a spectral image (1^st^ order). Next, we generated an identical spectral image (-1^st^ order). We modeled various noise sources, such as signal and background shot noise, and readout noise. The shot and readout noises follow Poisson and Gaussian distributions, respectively. Finally, we generated noise-added spectral images at different signal and noise levels^4^. We used a readout noise of 1 e- and 3000 iterations in all simulations.

We estimated the spatial precision of SDsSMLM using the simulated spectral images. The spatial precision was calculated using the standard deviation of the distribution of the estimated (*x*_0_, *y*_0_) in the virtual spatial image (Fig. 1e). By averaging the two spatial precisions along the x- and y- axes, we calculated a final spatial precision^7^. In addition, we estimated the spectral precision of SDsSMLM using the simulated spectral images. The spectral precision was calculated using the standard deviation from the distribution of the spectral centroid $\lambda_{\mathrm{SC}}$.

To compare the performance of SDsSMLM with that of sSMLM, we also estimated the spatial precision in sSMLM. We first generated noise-added spatial images at different signal and noise levels. Then, we estimated the spatial precision using standard deviation from the spatial location distributions. Additionally, we estimated the spectral precision of sSMLM. This procedure was essentially the same as described for SDsSMLM, except that only one spectral image corresponding to the 1^st^ order was used to obtain the emission spectrum.

Finally, we compared the spatial and spectral precisions of SDsSMLM with those obtained from sSMLM given varying splitting ratios between the 0^th^ and 1^st^ orders. For fair comparisons, we assumed that SDsSMLM and sSMLM share the same total number of photons. For SDsSMLM, the total photons were split equally between the -1^st^ and 1^st^ orders while sSMLM varied splitting ratios between the 0^th^ and 1^st^ orders.

In fact, the 0^th^ order channel will not exist in SDsSMLM when we use an ideal phase grating that only diffracts photons into -1^st^ and 1^st^ orders. These two spectral channels are mirror images of each other. We combine the two spectral channels for spectral analysis and create a “virtual spatial channel” for spatial imaging. In this case, we can directly compare the “virtual spatial image” (calculated from the -1^st^ and 1^st^ orders) with the “real spatial image” (from the 0^th^ order). Therefore, we compared performances between SDsSMLM using only two channels (-1^st^ and 1^st^ orders) and sSMLM (which has only two channels, 0^th^ and 1^st^ orders).

**Supplementary Note 4. Influence of splitting photons into multiple channels on SNR**

We conducted analytical and numerical simulations to investigate the influence of splitting photons into multiple channels on the SNR and the spatial precision of SMLM. Specifically, the model considers all main sources of noises, including signal shot noise, background shot noise, and readout noise^4,8^. We compared two representative cases: 1) all the emitted photons being collected in single channel, and 2) photons being divided into 2 channels.

In the numerical simulation, we first generated a spatial image of a single molecule using a PSF, which was modeled as a 2D Gaussian function. Then, we added various noises, including the signal shot noise, the background shot noise, and the readout noise, to the spatial image. The shot noise and the readout noise follow Poisson distribution and Gaussian distribution, respectively^8^. All key parameters used in the simulation are listed in Table S1. Next, we localized a center location of the spatial image by fitting it with a 2D Gaussian function using a maximum likelihood estimator^9,10^ for 100 frames. Lastly, we estimated the spatial precision from its location distribution. The spatial precision under the imaging conditions was determined using the analytical solution model^10^,

|  | ${\Delta x}^{2}=\frac{{F_{n}^{2}\sigma}^{2}+{a^{2}}/{12}}{Nq}\left( 1+4\tau+\sqrt{\frac{2\tau}{1+4\tau}} \right)$, | (S2) |
| --- | --- | --- |

where $\tau={2\pi(N_{b}q+n_{\mathrm{ro}}^{2})(\sigma^{2}+a^{2}/12)}/{Nqa^{2}}$; $\sigma$ is the standard deviation of the Gaussian function (nm); $a$ is the back-projected pixel size (nm); $N$ is the number of detected photons; *q* is the quantum efficiency (%); $N_{b}$ is the number of background photons per pixel; $F_{n}$is the excess noise factor of the EMCCD camera (dimensionless); and $n_{\mathrm{ro}}$ is the readout noise (e- pixel^-1^). We iterated this process for different photon counts with fixed readout noise and background. It is noted that we used the background of 30 photons pixel^-1^, a typical level previously reported in our sSMLM imaging^3^.

For the two-channel case, we implemented the numerical simulation in the same manner as described above except that we generated two noised-added spatial images with split signal photons and background at a 1:1 ratio. And, we combined the two spatial images before estimating the center location, which reasonably mimicked the actual biplane imaging in SMLM or SDsSMLM that fully use all the collected photons for the image reconstruction.

In addition, in order to calculate SNR, we extracted the signal photons, the background photons, and the readout noise directly from the generated spatial image. Then, we calculated the SNR by^8^,

|  | $SNR= \frac{N_{\mathrm{peak}}q}{\sqrt{\left( N_{\mathrm{peak}}+N_{b} \right){qF}_{n}^{2}+n_{\mathrm{ro}}^{2}}}$, | (S3) |
| --- | --- | --- |

where $N_{\mathrm{peak}}$ is the peak signal at the center pixel of the PSF.

Fig. S10a shows theoretically achievable spatial precisions when the total number of emitted photons increases and Fig. S10b shows SNRs when the peak signal increases for one- and two-channel cases. As shown in Fig. S10, these two values in the one-channel case (red circle) and the two-channel case (black cross) agree with each other, both of which also agree with the analytical solution (blue curve). Clearly, splitting the photons into two channels will not affect the SNR due to the background, which originates from the sample and system properties. In contrast, the variance of the readout noise from the sensor will be doubled as the photons are being recorded in two channels. However, in a typical SMLM setting, the readout noise is typically at a level less than 1 e- pixel^-1^, which is much lower compared with the shot noise generated by the signal (10 – ~38.7 photons pixel^-1^) and the background (~5.5 photons pixel^-1^) (Table S1) and its influence on the spatial precision is usually negligible^4,8-10^. Thus, the SNR of the recorded image is dominated by the background from the sample and system property, which is insensitive to the splitting of photons into multiple channels.

Table S1. List of parameters used in the simulation

| Pixel size, *a* (nm) | Quantum efficiency, *q* (%) | Background, $N_{b}$ (photons pixel^-1^) | Readout noise, $n_{ro}$ (e^-^ pixel^-1^) | Corresponding background shot noise (photons pixel^-1^) | Corresponding signal shot noise* (photons pixel^-1^) | Standard deviation of the Gaussian function, $\sigma$ (nm) | $F_{n}$ |
| --- | --- | --- | --- | --- | --- | --- | --- |
| 150 | 90 | 30 | 1 | ~5.5 | 10 – ~38.7 | 117 | $\sqrt{2}$ |

*the signal shot noise (photons pixel^-1^) was estimated from the peak signal at the center pixel of PSF (5x5 pixels), of a range of 100 – 1500 photons, which corresponds to a range of 385 – 5800 in the total number of emitted photons.

Supplementary information accompanies the manuscript on the *Light: Science & Applications* website (http://www.nature.com/lsa)

**References**

1 Deschout, H. *et al*. Precisely and accurately localizing single emitters in fluorescence microscopy. *Nature Methods* **11**, 253-266 (2014).

2 Dong, B. *et al*. Super-resolution spectroscopic microscopy via photon localization. *Nature Communications* **7**, 12290 (2016).

3 Zhang, Y. *et al*. Multicolor super-resolution imaging using spectroscopic single-molecule localization microscopy with optimal spectral dispersion. *Applied Optics* **58**, 2248-2255 (2019).

4. Song, K. H. *et al*. Theoretical analysis of spectral precision in spectroscopic single-molecule localization microscopy. *Review of Scientific Instruments* **89**, 123703 (2018).

5 Song, K. H. *et al*. Three-dimensional biplane spectroscopic single-molecule localization microscopy. *Optica* **6**, 709-715 (2019).

6 Zhang, Z. *et al*. Ultrahigh-throughput single-molecule spectroscopy and spectrally resolved super-resolution microscopy. *Nature Methods* **12**, 935-938 (2015).

7 Long, F., Zeng, S. Q. & Huang, Z. L. Effects of fixed pattern noise on single molecule localization microscopy. *Physical Chemistry Chemical Physics* **16**, 21586-21594 (2014).

8 Long, F., Zeng, S. Q. & Huang, Z. L. Localization-based super-resolution microscopy with an sCMOS camera Part II: Experimental methodology for comparing sCMOS with EMCCD cameras. *Optics Express* **20**, 17741-17759 (2012)*.*

9 Mortenson, K. I. *et al*., Optimized localization analysis for single-molecule tracking and super-resolution microscopy. *Nature Methods* **7**, 377-381 (2010).

10 Rieger, B. & Stallinga, S. The lateral and axial localization uncertainty in super-resolution light microscopy. *ChemPhysChem* **15**, 664-670 (2014).
